# A Voltage-Based STDP Rule Combined with Fast BCM-Like Metaplasticity Accounts for LTP and Concurrent “Heterosynaptic” LTD in the Dentate Gyrus *In Vivo*

**DOI:** 10.1371/journal.pcbi.1004588

**Published:** 2015-11-06

**Authors:** Peter Jedlicka, Lubica Benuskova, Wickliffe C. Abraham

**Affiliations:** 1 Institute of Clinical Neuroanatomy, Neuroscience Center, Goethe University Frankfurt, Frankfurt, Germany; 2 Department of Computer Science, University of Otago, Dunedin, New Zealand; 3 Brain Health Research Centre and Brain Research New Zealand, University of Otago, Dunedin, New Zealand; 4 Department of Psychology, University of Otago, Dunedin, New Zealand; Research Center Jülich, GERMANY

## Abstract

Long-term potentiation (LTP) and long-term depression (LTD) are widely accepted to be synaptic mechanisms involved in learning and memory. It remains uncertain, however, which particular activity rules are utilized by hippocampal neurons to induce LTP and LTD in behaving animals. Recent experiments in the dentate gyrus of freely moving rats revealed an unexpected pattern of LTP and LTD from high-frequency perforant path stimulation. While 400 Hz theta-burst stimulation (400-TBS) and 400 Hz delta-burst stimulation (400-DBS) elicited substantial LTP of the tetanized medial path input and, concurrently, LTD of the non-tetanized lateral path input, 100 Hz theta-burst stimulation (100-TBS, a normally efficient LTP protocol for *in vitro* preparations) produced only weak LTP and concurrent LTD. Here we show in a biophysically realistic compartmental granule cell model that this pattern of results can be accounted for by a voltage-based spike-timing-dependent plasticity (STDP) rule combined with a relatively fast Bienenstock-Cooper-Munro (BCM)-like homeostatic metaplasticity rule, all on a background of ongoing spontaneous activity in the input fibers. Our results suggest that, at least for dentate granule cells, the interplay of STDP-BCM plasticity rules and ongoing pre- and postsynaptic background activity determines not only the degree of input-specific LTP elicited by various plasticity-inducing protocols, but also the degree of associated LTD in neighboring non-tetanized inputs, as generated by the ongoing constitutive activity at these synapses.

## Author Summary

The vast majority of computational studies that model synaptic plasticity neglect the fact that *in vivo* neurons exhibit an ongoing spontaneous spiking which affects the dynamics of synaptic changes. Here we study how key components of learning mechanisms in the brain, namely spike timing-dependent plasticity and metaplasticity, interact with spontaneous activity in the input pathways of the neuron. Using biologically realistic simulations we show that ongoing background activity is a key determinant of the degree of long-term potentiation and long-term depression of synaptic transmission between nerve cells in the hippocampus of freely moving animals. This work helps better understand the computational rules which drive synaptic plasticity *in vivo*.

## Introduction

Synaptic plasticity, i.e. long-lasting activity-dependent changes in synaptic transmission, is widely considered to be a critical neural mechanism for memory storage. Computational models are being increasingly used to understand the precise activity rules that govern the induction and persistence of synaptic plasticity. However, the most recent models of plasticity based on the precise timing of pre- and postsynaptic spikes (i.e., spike-timing-dependent plasticity or STDP) typically ignore the fact that *in vivo* [1, 2], and also sometimes *in vitro* [3], the studied neurons exhibit an ongoing spontaneous spiking with frequencies reaching up to 10 Hz that has the potential to profoundly influence plasticity outcomes.

The first synaptic plasticity theory that explicitly took into account ongoing neuronal activity was the BCM theory [4]. A key element of this BCM theory is a whole-cell variable termed the modification threshold, the tipping point at which the presynaptic activity either leads to long-term depression (LTD) or long-term potentiation (LTP) of synaptic efficacy. A second key element is the theory’s postulate that the average ongoing level of background activity dynamically sets the position of the LTD/LTP tipping point in such a way that potentiation is favored when background postsynaptic cell firing is low on average and, vice versa, depression is favored when the postsynaptic activity is high on average. The BCM model has been used to account for experimental findings of experience-evoked plasticity in the developing visual [4, 5] and adult somatosensory cortices *in vivo* [6, 7]. The proposal of a modifiable plasticity threshold foreshadowed the concept of metaplasticity [8], developed to account for the abundant experimental evidence that prior neural activity can change the state of neurons and synapses such that the outcome of future synaptic plasticity protocols is altered [9].

In contrast to the BCM model, the standard STDP rule and its numerous modifications [10] as they are applied in simulations do not currently take into account background activity. A first rigorous attempt to bridge STDP with the BCM theory was made by Izhikevich and Desai [11], who showed that nearest spike STDP interactions led to a fixed LTD/LTP frequency threshold. However, according to the BCM theory, the position of the LTD/LTP threshold is not fixed but depends on the average postsynaptic activity (which is in turn proportional to the average presynaptic input activity). Thus, in the spirit of metaplasticity and the BCM theory, we have proposed that the magnitudes of LTP and LTD in the STDP rule are not constant but dynamically change from moment to moment as a function of the previous average postsynaptic spiking [12]. This line of thinking is in accordance with recent phenomenological equations for synaptic plasticity that use a rapidly changing LTD amplitude [13–15] or LTD window [16] based on average postsynaptic activity.

Granule cells in the hippocampal dentate gyrus have the intriguing ability to exhibit heterosynaptic plasticity when studied *in vivo*. Here, high-frequency stimulation (tetanization) of one set of synapses leads not only to LTP in that tetanized input but also LTD in a neighboring non-tetanized set of synapses [17–20]. We brought together the concepts of metaplasticity (effectively a fast BCM-like homeostasis) and STDP into a unified theoretical framework to model this as yet unexplained heterosynaptic plasticity phenomenon in the dentate gyrus of freely moving rats [12]. In that model, homosynaptic LTP occurred as a consequence of tetanization delivered to the medial perforant path input to a dentate granule cell, and LTD appeared simultaneously in the neighboring lateral perforant path synapses, as in experiments, but only if the model included ongoing spontaneous spiking for the lateral path inputs to drive the LTD mechanism under conditions of a transiently altered modification threshold that favored LTD. A direct prediction for experiments from this model was that blocking the spontaneous spiking of the lateral perforant path would also block the LTD. This prediction was tested and confirmed in anesthetized rats [21]. Based on the same STDP-BCM plasticity model, we were able to account for the absence of LTD using low-frequency stimulation paradigms in the dentate gyrus *in vivo* [22].

The above-mentioned computational studies used a highly simplified model of a dentate gyrus granule cell innervated by two representative excitatory synapses, one for medial path and one for lateral path, and modeled using the Izhikevich simple spiking neuron with parameters corresponding to a regularly spiking neuron [23, 24]. This model does not have dendrites, and thus we refer to it as a “point model” of a neuron. In spite of the successful account of the STDP-BCM plasticity model in modeling complex homo- and heterosynaptic plasticity phenomena in the dentate gyrus, important questions remain. Namely, will the plasticity model work also for a more biophysically realistic model of the granule cell with dendrites, featuring kinetics of all relevant membrane ion channels and multiple input synapses? Moreover, will our plasticity rule be able to reproduce the observed differential experimental effects of varying the frequency and temporal patterns of perforant path tetanization on the magnitude of synaptic plasticity? Here, we will pay particular attention to a recent experimental study of freely moving rats [25], in which different tetanization frequencies and temporal patterns applied to the medial path led to surprisingly different magnitudes of medial path LTP and, concurrently, LTD in the lateral path. Thus, although a 400 Hz delta-burst stimulation (400-DBS) administered to the medial path produced robust LTP and LTD, as previously described [20], unexpectedly the commonly used 100 Hz theta-burst stimulation (100-TBS) [26, 27] generated virtually no LTP or LTD. However, this theta-burst protocol could be converted into an LTP/LTD-inducing one by increasing the intraburst frequency from 100 to 400 Hz (400-TBS).

We show that our relatively simple STDP plasticity rule with fast BCM homeostasis / metaplasticity can reproduce the Bowden et al. [25] pattern of results when implemented in a compartmental granule cell model with realistic biophysics. The model thus gives insight into the computations that granule cells are making, as driven by plasticity-inducing synaptic events arising from both tetanization and background activity.

## Results

### Simulation of heterosynaptic plasticity in the compartmental neuron model

**Fig 1:**
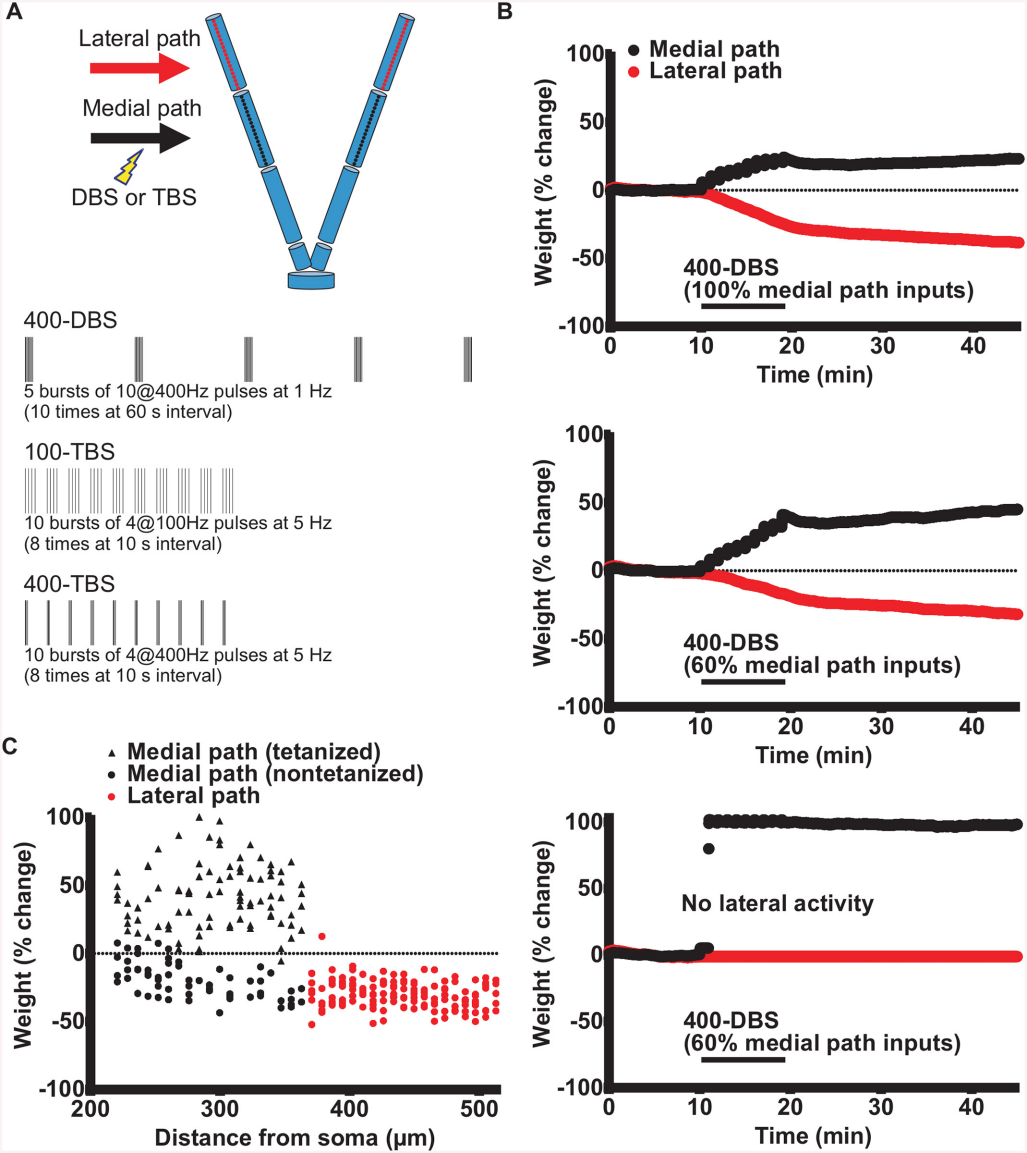
Biophysically realistic granule cell model for generating LTP on the medial path and concurrent LTD on the lateral path. (A) Top: Compartmental model of a granule cell with reduced morphology as introduced by Aradi and Holmes [28] and adapted by Santhakumar et al. [29]. See Methods for details. Red and black dots on the dendrites represent positions for medial and lateral perforant path synapses, respectively. (A) Bottom: Three HFS protocols applied to the medial path were used to test for LTP on the medial path and concurrent LTD on the lateral path: 400-DBS, 100-TBS and 400-TBS [25]. (B) Perforant path synaptic weights on a simulated granule cell before, during and after 400-DBS of the medial perforant path. Top: 400-DBS delivered to all medial path synapses produced smaller LTP on the medial path than concurrent LTD on the lateral path. Middle: 400-DBS delivered to 60% of medial path synapses produced greater LTP than concurrent LTD. Bottom: There was a loss of lateral path LTD when ongoing activity in that pathway was set to zero after the onset of 400-DBS (no lateral activity), demonstrating the need for ongoing spontaneous synaptic activity to drive LTD in the non-tetanized synapses. All graphs depict average values for all given synapses over 3 runs. (C) The spatial distribution of synaptic weight changes for 400-DBS delivered to 60% of medial path synapses, showing that non-tetanized medial synapses exhibit LTD. Weight changes are expressed as%change with respect to their baseline value.

To simulate heterosynaptic plasticity with a more complex type of model neuron, we adopted a published, multicompartmental and biophysically realistic computational NEURON model of a dentate gyrus granule cell, albeit with reduced morphology (Fig 1A and Methods; [28, 29]). This nine-section, 125 compartment granule cell model received input from 150 medial path synapses and 150 lateral path synapses, distributed in appropriate zones across the two dendritic branches (Fig 1A). This model was endowed with a synaptic plasticity mechanism containing a presynaptically centered nearest-neighbor implementation of the STDP rule with fast homeostasis (metaplasticity), the same as used previously for the point model [12] and described in the Methods. The multicompartmental granule cell model included the biophysics of the main ion channels in the dendrites and soma. Action potentials were generated in the soma and back-propagated along the dendrites, both electrotonically as well as due to the action of dendritic sodium and calcium ion channels. Thus the granule cell model took into account all of the complex spatio-temporal integration of EPSPs in the dendrites that are evoked by the spontaneous activity, the experimental high-frequency stimulation (HFS) protocol, and the back-propagating postsynaptic spikes. In addition, we employed a realistic simulation of the granule cell spontaneous input activity according to *in vivo* data that show a significant peak around 8 Hz for both the medial and lateral pathways [2]. For the initial series of simulations, the dendritic voltage threshold for the postsynaptic event detection at a synapse for local STDP implementation was -37 mV. To implement the global metaplasticity mechanism, the somatic voltage threshold for action potential detection for BCM homeostasis calculations was 0 mV.

### 400 Hz DBS

First, we modelled the 400-DBS protocol (Fig 1A) that has been used in many experiments for reliably inducing LTP and concurrent LTD [20, 25]. This protocol was implemented together with simulated ongoing presynaptic spontaneous activity at the mean rate of 8 Hz, phase-locked on average for the medial and lateral path synapses. In contrast to the point model, however, we introduced a random jitter for the presynaptic inputs (noise parameter = 0.05). Thus, each synapse received presynaptic spikes at slightly different times from other synapses, which is a more realistic account of the ongoing spontaneous activity *in vivo*. In another difference from the point-neuron model [12], we made no assumption regarding HFS affecting the ongoing presynaptic firing. Thus the presynaptic spontaneous activity pattern was maintained throughout the whole course of simulation: before, during and after HFS. Fig 1B (top panel) illustrates the evolution of mean medial path and lateral path weights in response to 400-DBS of all 150 medial path synapses for an optimal set of STDP parameters, i.e. A_p_ = 0.003, A_d_ = 0.001, τ_p_ = 20 ms and τ_d_ = 70 ms, α = 2500 (see Methods). This protocol generated robust LTP (23.0 ± 2.3%, n = 3 simulations) at the medial path synapses and even stronger LTD (-38.5 ± 1.3%) concurrently at the lateral path synapses. To make the model even more comparable to experiments, we also generated simulations whereby only 60% of the medial path synapses received the 400-DBS protocol. This latter choice was made because we considered it likely that in real-life experiments the electrical stimulation only engages a proportion of the input axons for a given pathway. The synapses chosen were randomly distributed spatially along the dendritic termination zone of the medial path inputs, and the same STDP parameters were employed. As shown in Fig 1B (middle panel), this protocol again gave a robust LTP at the tetanized synapses (45.3 ± 4.4%, n = 3 simulations) at the medial path synapses and now weaker albeit still robust LTD (-30.6 ± 1.5%) at the lateral path synapses. To investigate the relationship between localization of the synapse and magnitude of plastic change, the spatial distribution of the final weight for each of the 300 total synapses from a single simulation is plotted in Fig 1C. Nearly all synapses that received only the spontaneous activity showed LTD, whereas medial path synapses that received HFS potentiated. The degree of LTP or LTD did not depend on the distance from the soma (Pearson’s correlation coefficient r < 0.05).

It is noticeable that when 100% of the medial path synapses received HFS, the LTD had a larger absolute magnitude than the LTP. This is because the depressed lateral path synapses lie more distally on the granule cell dendrites than the medial path synapses, and due to greater electrotonic decay of the voltage from these distal synapses, the absolute change in synaptic weight needs to be greater than for the proximal potentiated synapses if a homeostatic restoration of firing rate to baseline levels is to be achieved. On the other hand, when only 60% of the medial path synapses were tetanized, not only all of the lateral path synapses but also the remaining 40% non-tetanized medial path synapses were depressed. With more total synapses being depressed, then the absolute average degree of depression of lateral path synapses could be less than the absolute average potentiation of the tetanized synapses and still maintain homeostasis. The relative balance of LTP and LTD in the 60% tetanization condition fits well with experimental observations [20, 25]. When the number of tetanized medial path synapses was reduced to only 20%, however, the balance between LTP and LTD shifted too far in favour of LTP, compared to the experimental data (S1 Fig).

It is worth noting that, as predicted by the point model and shown in experiments [21], lateral path LTD was absent when spontaneous activity on this pathway was turned off during and after medial path HFS (Fig 1B, bottom panel). This is because the LTD only occurs in the model when the STDP rule is triggered by lateral path ongoing spontaneous activity.

### 100-TBS

**Fig 2:**
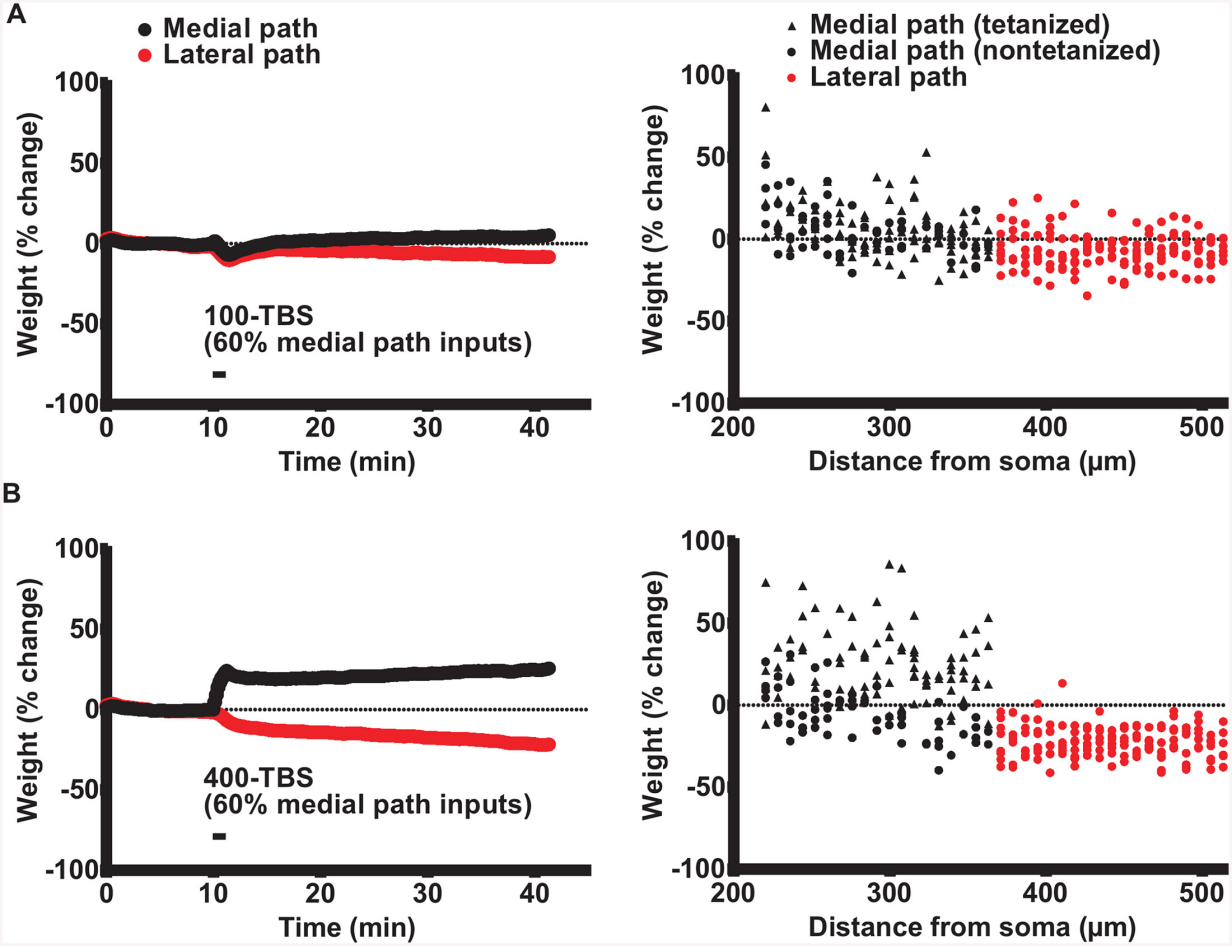
LTP and concurrent LTD evoked by 100-TBS and 400-TBS applied to the medial path. (A) 100-TBS (B) 400-TBS. Left: Evolution of medial and lateral path synaptic weights in computer simulations expressed as % change with respect to their baseline value. Averages are of 3 runs. Right: The corresponding synaptic weight changes versus distance from soma. Note that 400 Hz protocol produced greater LTP and concurrent LTD than the100 Hz protocol, as observed in experiments of Bowden et al. (2012) [25].

We next tested whether the ineffectiveness of the 100-TBS protocol (Fig 1A) in eliciting synaptic plasticity in experiments could be reproduced in the compartmental model. Indeed, when using the same parameters as optimal for the DBS protocol, this TBS protocol produced only weak LTP (5.3 ± 0.3%, n = 3) of the tetanized medial path synapses (60% tetanization protocol) and an equally weak concurrent LTD of the non-tetanized lateral path synapses (-6.5 ± 0.3%, Fig 2A). These changes were much smaller in magnitude than for 400-DBS, as observed in experiments [25]. Similar effects were seen when 100% of the medial path synapses were tetanized.

### 400-TBS

In the experiments, LTP and concurrent LTD induction could be largely rescued simply by increasing the pulse frequency within a theta-burst from 100 to 400 Hz, keeping all other parameters the same. Accordingly, we assessed the synaptic weight evolution for the 400-TBS protocol in the compartmental model. We observed a recovery of LTP for the tetanized medial path synapses and of LTD for the non-tetanized lateral path and medial path synapses, when tetanizing 60% the medial path synapses (Fig 2B). The 400-TBS protocol produced mild LTP (25.0 ± 2%, n = 3) of the tetanized medial path synapses (60% tetanization protocol) and mild concurrent LTD of the non-tetanized lateral path synapses (-20.7 ± 1.6%), as seen in experiments [25]. Similar effects were seen when tetanizing 100% of the medial path synapses.

### Summary of results and robustness of the model

**Fig 3:**
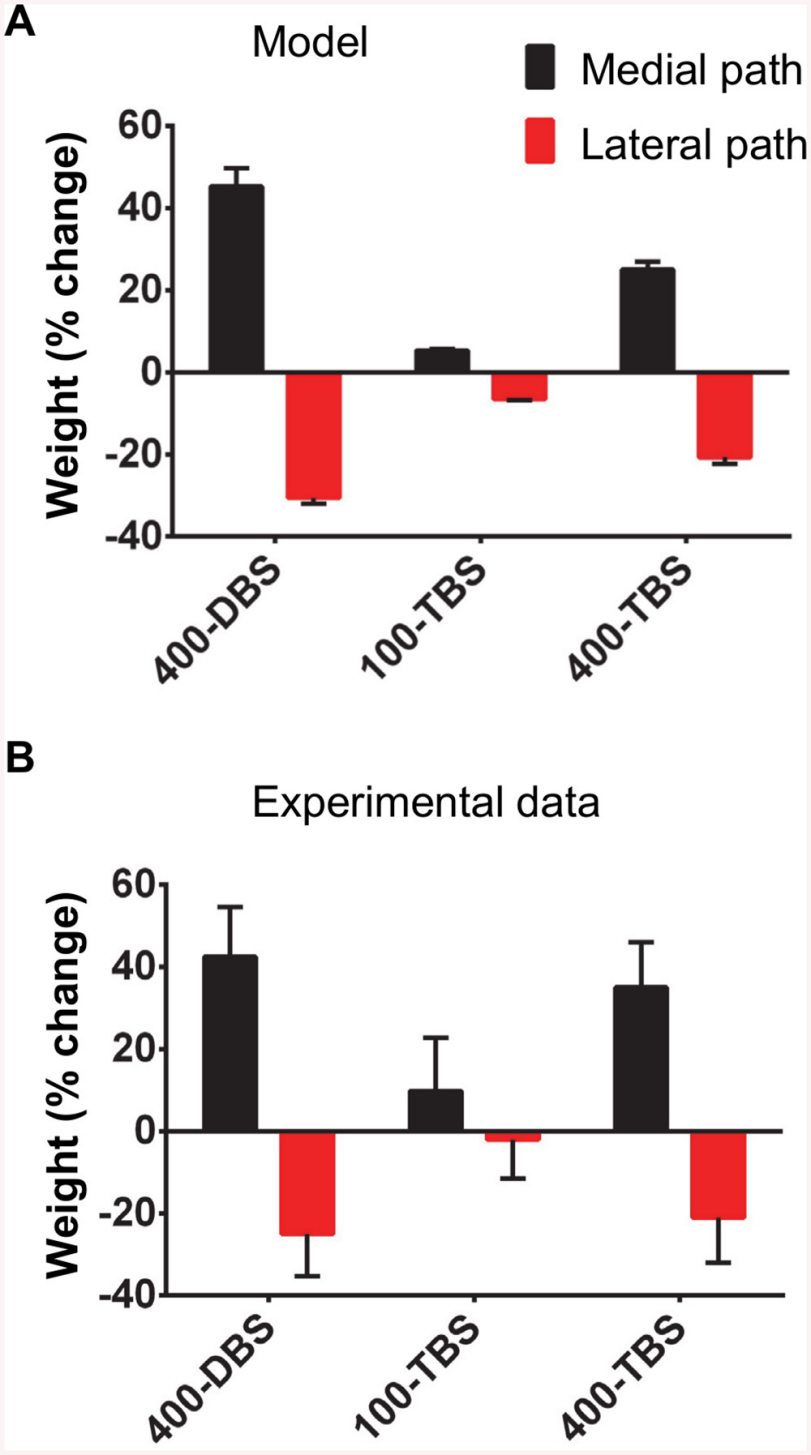
Summary of effects of stimulation pattern on LTP and concurrent LTD in compartmental model simulations and experiments. 400-DBS and 400-TBS produced greater LTP and concurrent LTD than 100-TBS; (A) *in silico* for the optimal values of parameters (average results from 3 runs); (B) *in vivo* (Bowden et al. 2012, [25]).

Fig 3 summarizes the plasticity results (60% medial path tetanization) using the compartmental model, and compares them with the experimental data obtained by Bowden et al. [25], using the parameter set described above. It is important to consider, however, whether the qualitative or quantitative match with the experimental data is dependent on these specific parameters, or whether the STDP-BCM model has more generality. Accordingly we systematically varied the level of noise, τ_d_, τ_p_, the initial ratio of A_p_ / A_d_ and the dendritic threshold voltage for detecting the postsynaptic event for STDP pairing. The results are given as averages from 10 runs (± S.D.). To speed up these simulations, we reduced number of segments to 9, i.e. 1 for soma, 2 for the granule cell layer, 2 for the inner molecular layer, 2 for the middle molecular layer and 2 for outer molecular layer parts of the dendrites.

First we investigated the effect of the level of noise in the ongoing input spontaneous spiking. Noise = 0 means perfectly synchronized 8 Hz spiking at all synapses, while Noise = 1 means all synapses receive random, Poisson-distributed spiking with an average frequency of 8 Hz. Noise values ranging from 0.05 to 1.0 all provided excellent matches with the experimental data (S2 Fig). For the case of Noise = 0.01 and 0, the two pathways either both potentiated or both depressed, but this could be prevented by adjusting the starting conductances for both the medial and lateral path synapses. Overall, the model was very robust with respect to the asynchrony of the ongoing background activity. Intriguingly, the model performed better in the presence of noise than in the absence of it, even when there was completely random synaptic activity.

Also of interest was the dependence of the model on time constant values (τ_p_ and τ_d_) for the plasticity windows, as in experiments they can vary substantially across brain regions, cell types and the preparations used [30]. As commonly observed experimentally, we found that τ_d_ needed to be somewhat greater than τ_p_, with the best match to the experimental data seen with τ_d_ = 70 ms and τ_p_ = 20 ms (S3 Fig). Quantitatively poor results were obtained with pairings of 30/20 ms and 70/40 ms (τ_d_ and τ_p_, respectively), although qualitatively the patterns of LTP and LTD outcomes across the three HFS conditions remained similar across the parameter values used (S3 Fig). For the optimal pairing of τ_d_ = 70 ms and τ_p_ = 20, we also found that the starting amplitudes of potentiation (A_p_) and depression (A_d_) in a 3:1 ratio gave a result matching the experimental data, while a ratio of 1:1 gave a qualitatively and quantitatively poor match (S4 Fig).

As a final test of model robustness, we compared the effects of various synaptic voltage thresholds for triggering the STDP calculations, specifically -30 mV, -33 mV, -37 mV, and -40 mV. We found that -40 mV gave a very similar result to that obtained with our standard -37 mV threshold, but that setting the threshold at -30 mV distorted the outcome relative to the experiments (S5 Fig), as it was common for the back-propagating spike to fail to reach this threshold for the bulk of the lateral path synapses. Taking the results of all these simulations together, however, the BCM-STDP plasticity rule was robust across a wide range of parameters in its ability to reproduce the experimental data, when implemented in a multicompartmental model of a granule cell.

### Mechanisms regulating plasticity induction

**Fig 4:**
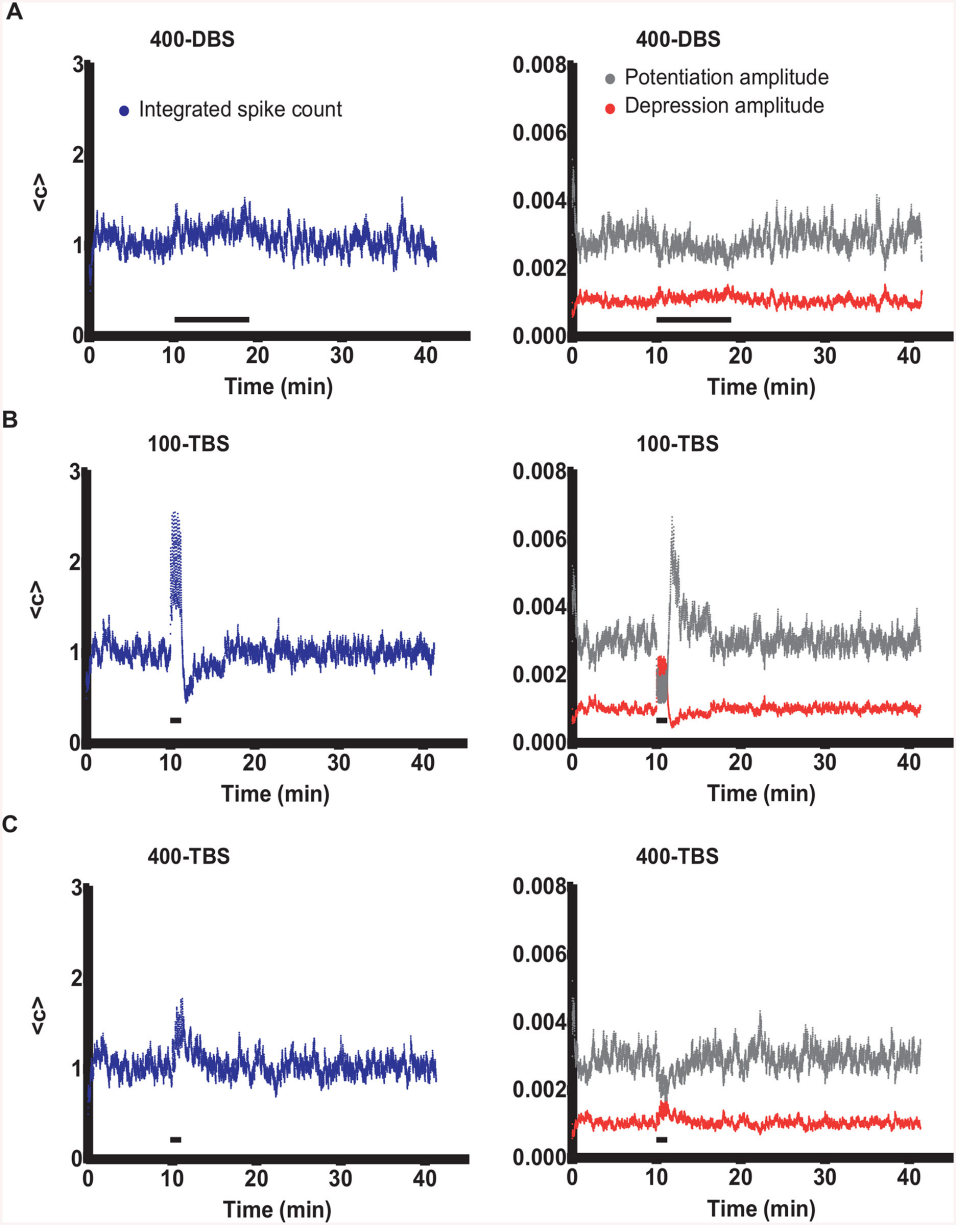
Granule cell firing in response to HFS and associated homeostatic adjustment of LTP and LTD amplitudes before, during (horizontal black bars) and after HFS. (A) The integrated spike count and thus also potentiation as well as depression amplitudes (A_p_, A_d_) remained steady during 400-DBS. (B) 100-TBS generated more spikes during the HFS than the DBS protocol (see also Fig 5). In response to the increased spike count during 100-TBS, potentiation and depression amplitudes were rapidly and strongly decreased and increased, respectively. (C) 400-TBS generated fewer spikes than 100-TBS (see Fig 5) resulting in smaller homeostatic changes in potentiation and depression amplitude. See text for details.

**Fig 5:**
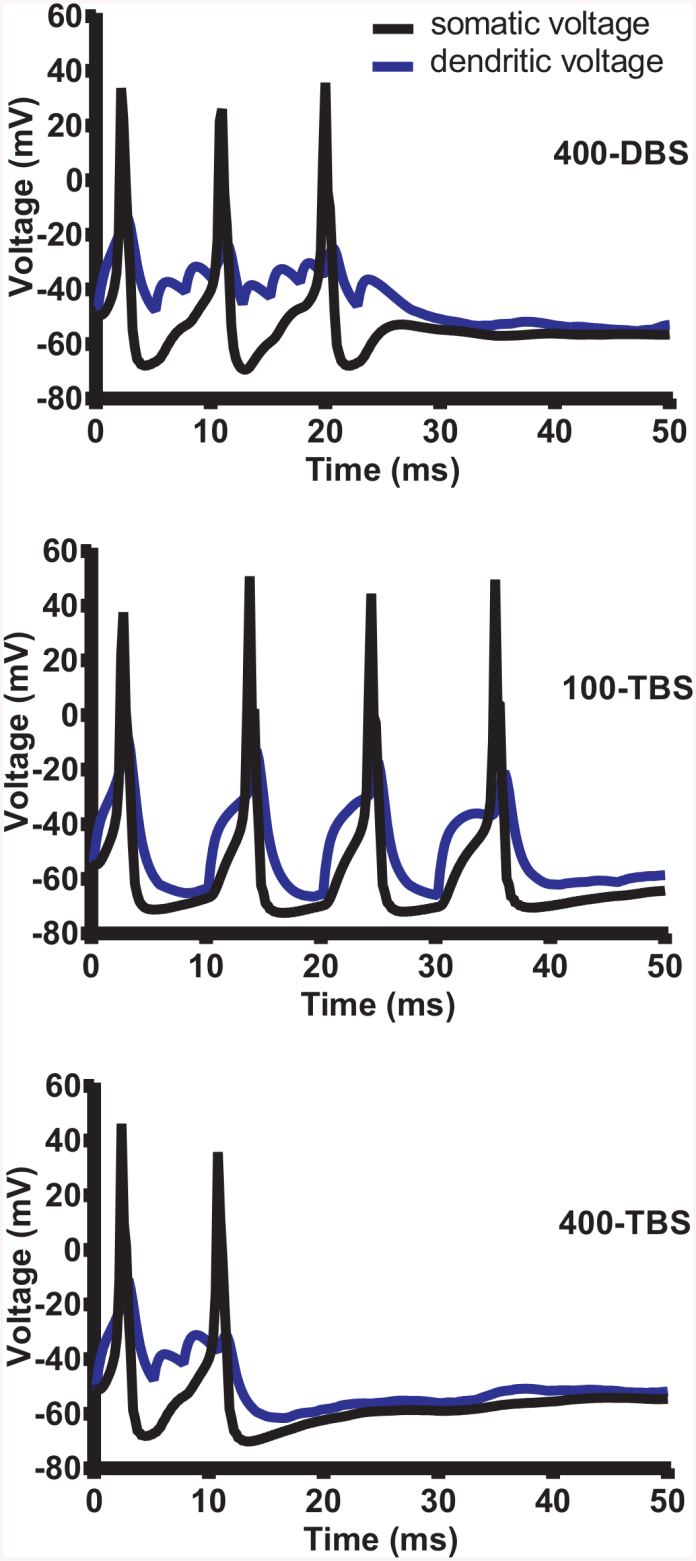
Postsynaptic membrane voltage during one train of HFS. Simulated voltage traces showing the depolarization and action potentials evoked by 400-DBS (top panel), 100-TBS (middle panel) and 400-TBS (bottom panel) and measured at the soma (black trace) and the dendrite (blue trace). The dendritic voltage was recorded in the middle of the dendritic region targeted by medial path synapses. Note the faithfulness of cell firing for each TBS pulse for the 100-TBS protocol. In simulations of LTP and LTD, the postsynaptic threshold for STDP was set to -37 mV so that STDP could be exhibited by even the most distal of synapses on the dendritic tree.

**Fig 6:**
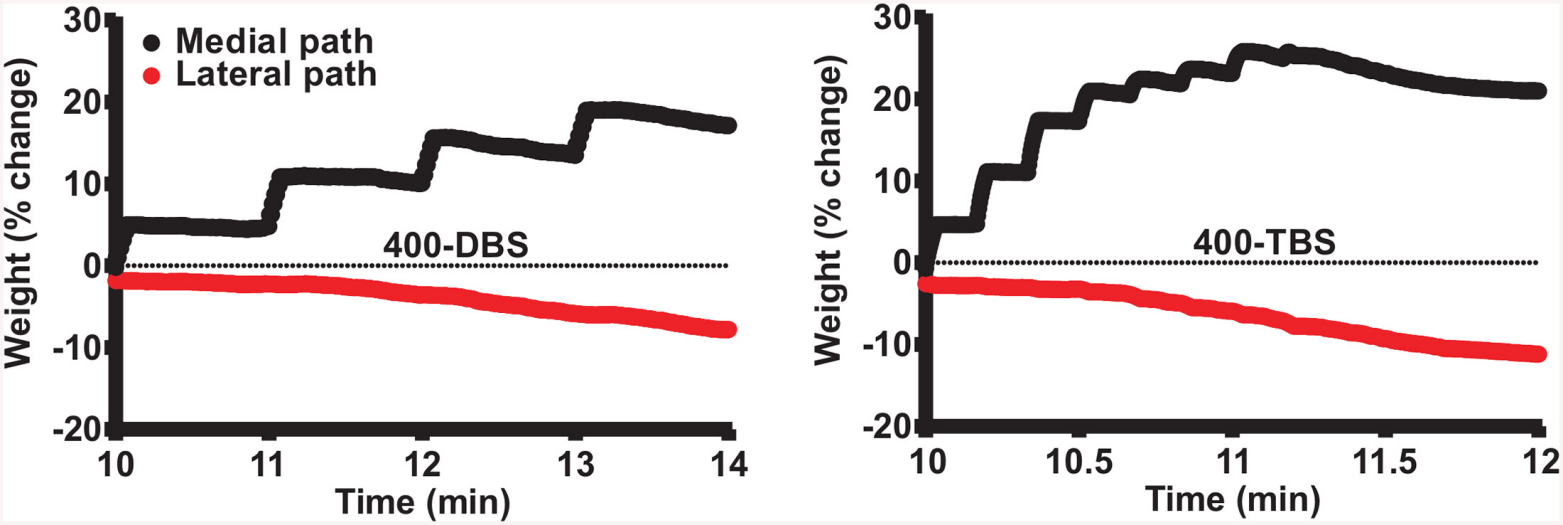
Evolution of medial and lateral path synaptic weights early in the HFS period. Note that the initial increase in the medial path response occurs before the decrease in lateral path response indicating that, because of homeostatic nature of the model, LTP drives LTD. Left panel, 400-DBS; right panel, 400-TBS.

The multicompartmental model of granule cell, with realistic active and passive properties, was able to reproduce different experimental results arising from the various experimental protocols. This afforded us the opportunity to examine the firing properties of the granule cell model in response to HFS in order to ascertain biophysical explanations for the protocol dependence of the different plasticity outcomes. Of particular interest to us was the integration of postsynaptic action potential firing, which is the key parameter for BCM-based adjustment of the STDP parameters A_p_ (potentiation-amplitude) and A_d_ (depression-amplitude). The integrated spike count 〈c〉 (factored by a constant α) for the first protocol, 400-DBS, is shown in the left panel of Fig 4A. The right panel shows the corresponding dynamic changes in A_p_ and A_d_ as these latter parameters are scaled by 〈c〉. For this protocol, after the initial period of stabilization, time-averaged 〈c〉 remained steady and was only mildly increased during HFS of the medial path, because each burst of 10 presynaptic pulses (at 400 Hz) generated only 2–4 postsynaptic spikes due to action potential refractoriness (Fig 5, upper panel), and therefore both A_p_ and A_d_ barely changed. Thus it can be concluded that the DBS protocol was sufficient to instigate LTP of the medial path using relatively unchanged STDP parameters. Close inspection of the development of LTP and LTD shows that LTD began to arise only after LTP had begun to develop (Fig 6), indicating that the LTD in the lateral path was occurring as a consequence of LTP induction in the medial path. Lateral synapses were depressed because the spontaneous activity of the potentiated medial path started controlling postsynaptic spiking, and so the lateral path activity was participating less in the LTP-inducing pre-post order than in the LTD-inducing post-pre order.

When we examined the spiking response to the 100-TBS paradigm, the time-averaged 〈c〉 was greatly increased during tetanization (Fig 4B). Close inspection of the voltage traces (Fig 5, middle panel) showed that for this protocol, almost every presynaptic volley caused a postsynaptic spike. Thus, even though this protocol has only 4 pulses per burst, by spiking the postsynaptic cell 4 times for each burst, this protocol actually generated more spikes than the DBS protocol. And since the bursts also come more frequently (5 Hz) than for DBS (1 Hz), the integrated spike count rises even more dramatically. Surprisingly, despite this increase in postsynaptic spiking, LTP induction was very weak using the 100-TBS protocol. This counterintuitive result is explained by the fast homeostatic nature of our learning rule which generates a rapid and dramatic decrease in A_p_ and corresponding increase in A_d_ in response to the increased spiking (Fig 4B), and these effects together considerably braked the induction of LTP in the medial path. The increase in A_d_ does not lead to a larger LTD, however, because its induction occurs mainly to balance the degree of LTP induction. To confirm that the change in A_p_ contributes to braking LTP, we turned off the homeostatic adjustment of A_p_, and found that the average degree of LTP indeed increased from 5.3 ± 0.3% to 36.8 ± 0.9%, as predicted.

If the enhanced spiking during 100-TBS, and resultant change in A_p_ and A_d_, were responsible for observing less LTP compared to 400-DBS, than we can predict that LTP should be rescued for the theta-burst protocol when the intra-burst frequency is increased from 100 Hz to 400 Hz. This should reduce somatic spiking due to action potential refractoriness, reduce 〈c〉, and thus dampen the homeostatic changes in A_p_ and A_d_. This is exactly what happened for the 400-TBS protocol (Figs 4C and 5, bottom panel).

### The need for active back-propagation of the action potential

**Fig 7:**
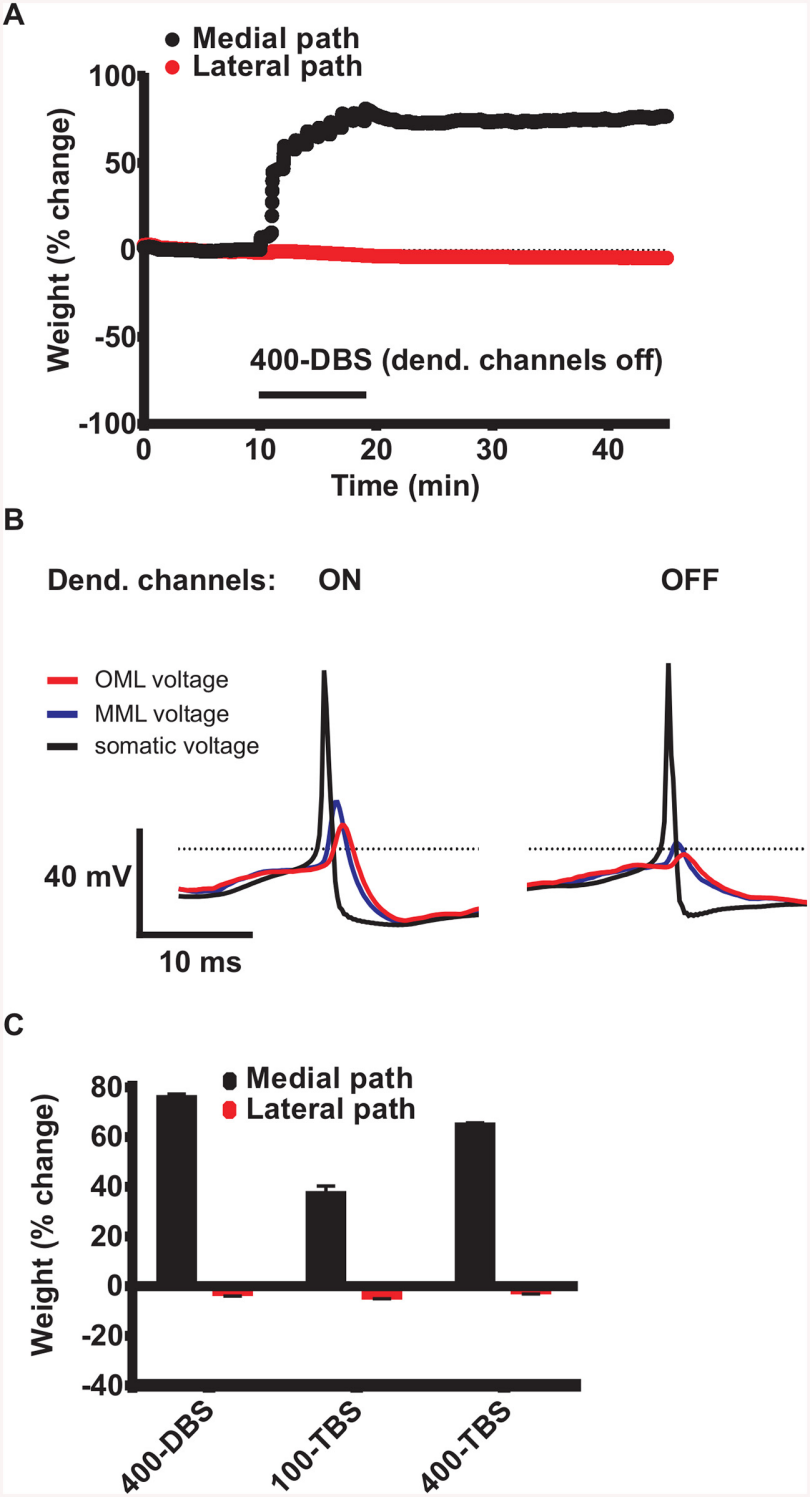
Active back-propagation of the action potential is not needed forLTP at medial path inputs. To block active back-propagation of action potentials, all the voltage-dependent sodium and calcium channels in the dendrites were turned off at the beginning of the DBS. (A) Evolution of medial and lateral path synaptic weights before, during and after 400-DBS of the medial path. (B) Simulated voltage traces showing sample action potentials evoked by spontaneous synaptic activity before (left panel) and after switching off the channels (right panel). Voltage was measured at the soma (black trace) and in dendrites in the middle molecular layer (MML, blue trace) and outer molecular layer (OML, red trace). MML and OML are targeted by medial and lateral perforant path synapses, respectively. Note that when active channels were switched off, action potential failed to cross the threshold for STDP in the OML. (C) Summary of effects of stimulation pattern on LTP and concurrent LTD in simulations with the blockage of active channels in the dendrite. Note that active propagation was necessary for the triggering of LTD in the lateral path but not for LTP in the medial path.

The dentate granule cell is electronically compact, and therefore it is not clear whether active back-propagation of the action potential is needed for all the required STDP interactions to take place, especially for the lateral path synapses located on the distal dendrites. To address this issue, we turned off all the voltage-dependent sodium and calcium channels in the dendrites, and ran our model with the standard parameter set (60% medial path HFS) for the three tetanization protocols. For the medial path synapses, the degree of LTP was increased for all three protocols, but the qualitative relations between them was preserved (Fig 7). STDP (and LTP) occurs in the MPP synapses without action potential backpropagation (when dendritic sodium and calcium channels are blocked) because DBS and TBS induced sustained depolarization through electrotonic conduction which is able to cross the local plasticity threshold (Fig 7A and 7B). This is in line with recent experimental observations of LTP induction in the absence of somatic action potentials ([31], see also [32]). Similarly, turning off somatic sodium channels during HFS did not prevent LTP induction but it worsened the match with experiments (the model failed to produce balanced LTP and LTD) since the metaplasticity mechanism in the model needs somatic spikes to get activated. In contrast to LTP, when dendritic channels were inactivated, no significant LTD appeared on the lateral path synapses for any of the HFS protocols (7C). This can be explained by the failure of the passively conducted APs to breach the -37 mV threshold for STDP in the outer molecular layer, in contrast to what happens in the middle molecular layer (Fig 7B). Thus, some degree of active propagation is necessary for the triggering of STDP in the lateral path synapses, at least for the voltage threshold for STDP that we have used.

### The need for a fast Bienenstock-Cooper-Munro (BCM)-like homeostasis / metaplasticity rule

**Fig 8:**
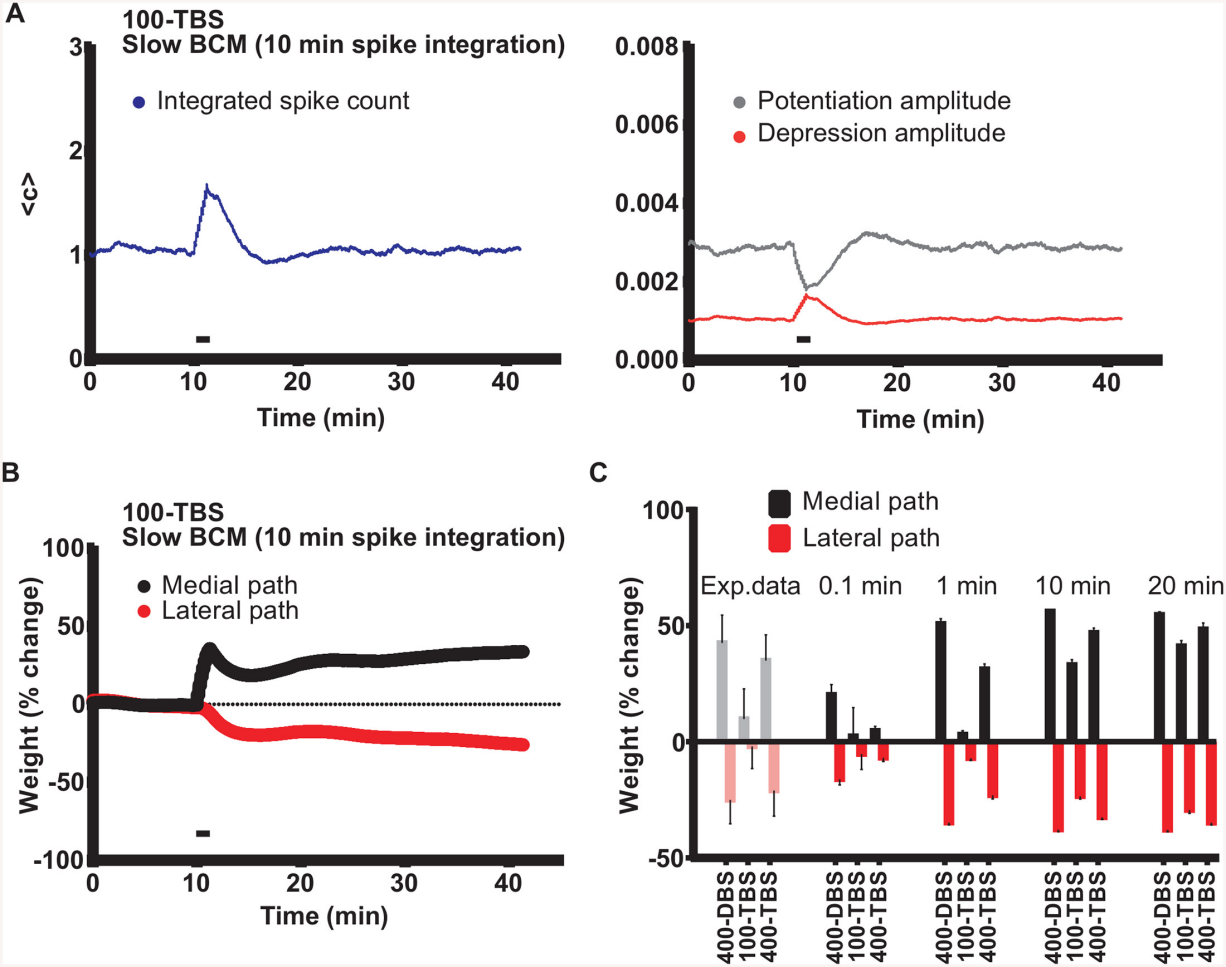
A fast BCM-like homeostasis/metaplasticity rule reproduces experimental data better than slow BCM-like rules. (A) Evolution of the integrated spike count (left) and potentiation/depression amplitudes (right) during an LTP/LTD simulation using slow BCM-like homeostasis (10 min integration period). 100-TBS was delivered to the medial path. Note that changes of spike count and potentiation/depression amplitudes were much less pronounced as compared to simulations using our standard 1 min integration period (see Fig 4B). (B) LTP and concurrent LTD evoked by 100-TBS applied to the medial path, using the 10 min integration period. Note the enhanced LTP and LTD from this simulation. (C) Summary of effects of stimulation pattern on LTP and concurrent LTD in simulations with integration periods ranging from 0.1 to 20 min. Only simulation using the 1 min integration period accurately reproduced the experimental data.

Recent theoretical results indicate that stable activity in neural networks requires a homeostatic rule with a fast detection (seconds to minutes) of firing rate changes [14]. Therefore, we wanted to test whether fast integration of spikes, which is a key part of our BCM-like metaplasticity rule, is needed to account for the experimentally determined pattern of LTP and LTD results. When we slowed down the integration of spikes from 1 min to 10 min, the degree of plasticity changes still occurred in the order 400-DBS > 400-TBS > 100-TBS, but the quantitative match with data from Bowden et al. [25] was much worse (Fig 8C). In particular, the 100-TBS protocol generated unrealistically large LTP and LTD (Fig 8B and 8C). The reason for this is that slow BCM homeostasis leads to a smaller rise in integrated spike count and thus a smaller decrease in A_p_ and a smaller increase in A_d_ in response to the increased spiking during 100-TBS (c.f. Figs 4B and 8A). The match was even worse for an integration time of 20 min, and also for the extremely fast integration time of 0.1 min (Fig 8C). We conclude that fast (but not too fast) BCM-like metaplasticity is important for best explaining the different plasticity outcomes from different HFS patterns.

## Discussion

In this study, we used computational modeling of plasticity at the medial and lateral perforant path inputs to dentate gyrus granule cells to account for the effects of different frequencies and temporal patterns of HFS on the induction of homosynaptic LTP and concurrent heterosynaptic LTD, as observed *in vivo* [25]. Importantly, the HFS protocols were superimposed on realistically simulated spontaneous spiking activity of the pre- and postsynaptic neurons because we believe that spontaneous spiking activity should be considered in theoretical models of synaptic plasticity (c.f. [33, 34]).

The main conclusions are: (1) Combined STDP and BCM rules can reproduce the LTP and heterosynaptic LTD, (2) as long as spontaneous activity continues in the input pathways. (3) The degree of LTD depends on the degree of LTP, due to the implemented homeostatic BCM rule that stabilizes cell firing rate. (4) Standard 100 Hz-TBS gives counter-intuitively poor LTP and LTD because this protocol is very good at firing granule cells, which in turn causes the potentiation amplitude parameter to transiently decline, hence braking LTP.

We have combined the ideas of STDP and BCM theory in such a manner that the timing between the presynaptic spike and postsynaptic events at the site of a synapse influences the sign of synaptic weight change; at the same time, the size of prospective LTP and LTD depends (in alignment with the BCM theory) on the average postsynaptic activity at the neuron’s soma over some recent past (on the order of seconds to minutes). Thus, the amplitude parameters for potentiation and depression are not constant but dynamically change; potentiation is increased when the average postsynaptic firing is low while the depression size is decreased, and the opposite is true when the average postsynaptic firing is high. This constitutes a robust homeostatic mechanism. The model proved to robustly reproduce the experimental data across a wide range of parameter settings, lending confidence to validity of the model’s assumptions.

### Importance of ongoing spontaneous activity for generating heterosynaptic LTD

Our biologically realistic simulations have shown that ongoing background activity is a key determinant of the degree of long-term potentiation and especially long-term depression in the dentate gyrus. All HFS protocols were implemented together with simulated ongoing presynaptic spontaneous activity at the mean rate of 8 Hz. This spontaneous spiking was phase-locked only on the average for the medial and lateral path synapses, as we introduced random jitter across a wide range for all the presynaptic inputs. Thus, each synapse received presynaptic spikes at somewhat different times from other synapses, which is a more realistic account of the spontaneous activity *in vivo*. This ongoing spontaneous input activity can explain why the heterosynaptic LTD is routinely seen in the dentate gyrus *in vivo*, when the hippocampal circuitry is intact [18–20] while absent in the dentate gyrus *in vitro*, when the input is severed [35]. We would like to point out that in neocortical slices too a substantial spontaneous activity can be present [3] and that may be why the heterosynaptic LTD can happen [36].

### LTP/LTD phenomenological mechanisms

To summarize the operation of the compartmental granule cell model, medial path HFS causes the granule cell to repeatedly spike, and due to the causal pre-post order, the medial path synapses thus potentiate. Then, as the strengthened medial path spontaneous activity begins to assume control of the cell spiking, the lateral path spontaneous activity moves more into the LTD zone of the STDP windows. Concurrently, the spiking caused by HFS homeostatically adjusts the depression amplitude upward for some time, enhancing LTD induction. While a concomitant drop in potentiation amplitude also occurs, this is not sufficient to prevent LTP induction in the medial path with 400 Hz protocols. However, the 100 Hz-TBS gives poor LTP and thus poor LTD because TBS is so good at firing granule cells that this causes the potentiation amplitude parameter to markedly decline, braking LTP induction. This happens due to the BCM part of our STDP rule which causes that the increased postsynaptic spiking (evoked by 100-TBS) leads to a decrease in the potentiation amplitude parameter A_p_ and this reduces the induction of LTP in the medial path. The difference in firing of the granule cell in combination with the homeostatic STDP-BCM plasticity rule is the main cellular mechanism which explains the difference in plasticity outcomes (400-DBS > 400-TBS > 100-TBS). The difference in firing arises due to action potential refractoriness which depends on sodium and potassium channels in the soma of the granule cell.

### Postsynaptic events for the STDP nearest neighbor interactions

In the published point model [12], the postsynaptic event setting the timing for STDP weight changes is the postsynaptic spike. In our new morphological (multicompartmental) model, it is the threshold dendritic voltage at any synapse location that would register the occurrence of a back-propagating action potential (bAP) for the purpose of STDP calculations. For most of the above simulations, we used -37 mV as that threshold membrane voltage, although other voltages could be used, as long as the distal dendrites could be driven to this threshold voltage by an action potential [37]. Thus STDP could be exhibited by the full extent of synapses on the dendritic tree [38, 39]. It is noteworthy however that the postsynaptic event can result from summation of the local EPSPs and/or any bAP crossing certain threshold value. Thus, it is an interesting aspect of using -37 mV as the local STDP threshold that sometimes this threshold was crossed by synchronous synaptic activity even in the absence of a somatic action potential. This was particularly true during 400 Hz bursts, when the soma could not follow the rate of presynaptic activity, but the synchrony of afferent input was sufficient for the synaptic response to cause the membrane potentiation to rise higher than -37 mV. The extent to which this influenced the degree of LTP and LTD is not clear. However, since LTD induction lagged behind the LTP induction, it remained the case that post-HFS back-propagation of the action potential from the soma was the critical dendritic event for at least these STDP calculations.

The necessity of having a postsynaptic threshold for a postsynaptic event in our model is in line with the hypothesis of Lisman and Spruston [40] that long-lasting synaptic modification may be protected by multiple thresholds that have to be crossed before weights can be persistently modified. First of these thresholds would be a critical dendritic depolarization that has to be reached as a result of temporal and/or spatial integration of EPSPs and back-propagating APs [40]. Kovalchuk et al. [41] showed that –30 mV is the voltage where the NMDA-mediated Ca^2+^ influx is maximal in the dendritic spines of CA1 cells. Since we have only one synaptic threshold for both LTD and LTP, a value slightly below -30 mV seems reasonable as a calcium increase is required also for induction of LTD, although we found -33 mV to be an effective threshold as well. Clopath et al.’s synaptic plasticity model [13] has two voltage thresholds, one for LTD and one for LTP, but the value they used for hippocampal cells were very similar, i.e. -41 mV and -38 mV, respectively.

### Fast homeostatic metaplasticity

A critical parameter of the model that affects BCM calculations is the length of the cell-firing integration period. Our model was the most stable and robust when using a relatively fast integration period of one minute. Here we briefly discuss a potential biochemical mechanism for this relatively fast homeostasis. When the amplitude of the postsynaptic Ca^2+^ signal falls above (or below) a certain threshold, then active synapses manifest LTP or LTD, respectively [34, 42]. Many intracellular proteins contribute to these processes, with Ca^2+^-calmodulin-dependent protein kinase II (CaMKII) being a key element in LTP induction and possibly also in LTD induction [43, 44]. But since CaMKII is generally inactivated in a relatively short timeframe (∼1 min) after transient synaptic stimulation, other biochemical pathways must play important roles in the maintenance LTP [45]. The timeframe of CaMKII inactivation (∼1 min) is particularly relevant to our putative homeostatic mechanism. This inactivation is caused by increased intracellular Ca^2+^ that leads to autophosphorylation of CaMKII, thereby converting the enzyme to a Ca^2+^-independent (autonomous) form. That is, even if there is a presynaptic signal that repeatedly opens NMDARs and further increases intracellular calcium, the relative magnitude of further LTP would be smaller than at the beginning of the synaptic stimulation. The opposite might be the case for the magnitude of LTD, because the enzymatic pathways leading to LTD are not inhibited anymore. Thus, we speculate that the level of Ca^2+^-independent CaMKII might be related to the fast dynamics of A_p_ and A_d_ (see also [33, 46]).

### Relevance to current data and models on metaplastic homeostasis

Metaplasticity is defined as the activity dependent and persistent change in neuronal state that shapes the direction, duration and/or magnitude of future synaptic change [8, 9]. A typical computational implementation of synaptic metaplasticity theory is the BCM-like model of synaptic plasticity in which the sign and magnitude of plasticity, as well as the position of the sliding modification threshold, are governed by the level of postsynaptic activity averaged over some past. The BCM-like sliding modification threshold serves a homeostatic function by producing cell-wide changes that keep synaptic plasticity within a working dynamic range and flexible. This has the net effect of keeping both LTP and LTD readily available to respond to future changes in correlated presynaptic and postsynaptic activity [47]. In our model, we implemented BCM-like metaplasticity, in which the metaplastic state affects all synapses across the cell. However we do not exclude other metaplastic mechanisms that take place on a semi-global or even local level [47].

Zenke et al. [14] carried out simulations of large balanced networks with a homeostatic triplet STDP rule, in which the timescale of homeostasis was on the order of seconds (from 3 to 25 s; see also [15]). The moving average of the postsynaptic spike count was used to dynamically adjust only the LTD amplitude. Thus in their simulations with metaplastic triplet STDP, the amount of LTD varied homeostatically as a function of the moving average of the postsynaptic firing rate (see also [13]). The difference in metaplastic equations between Zenke et al. [14] and our model is that our model assumes that the amplitude for LTP is also metaplastically regulated, as we do not find any experimental justification for why only the amplitude for LTD should be metaplastically modified and also metaplastic LTP and LTD amplitudes gave us a better homeostatic control.

### Summary and predictions

We have found that combining STDP and BCM-like rules with spontaneous activity can replicate the outcomes of three separate *in vivo* experiments in a biologically realistic (i.e. morphological) granule cell model. The new model is a significant improvement on the previous point neuron model [12], which required some unrealistic assumptions in order to fit the data. Models have value not only in helping to understand complex mechanisms that may be difficult to assess experimentally, but also generate predictions that feed back to the experiments for validation purposes, and further understanding of the biology. From our model, we can make the following experimentally testable predictions: 1) that blocking the dendritic voltage-dependent sodium and calcium channels during and after HFS will disrupt heterosynaptic LTD of lateral path synapses but leave homosynaptic medial path potentiation intact; 2) that fast (but not too fast) metaplasticity is necessary to achieve a good match with experimental data; 3) that spiking by the granule cells should be higher during 100-TBS than for the other two protocols; 4) that if HFS activates a higher percentage of medial path fibers, the average amplitude of medial path potentiation gets smaller but the average amplitude of lateral path depression gets larger; 5) that decreasing spontaneous activity will lead to stronger homosynaptic LTP but weaker heterosynaptic LTD; 6) that altering the history of granule cell firing will dynamically change the size of STDP windows for LTP and LTD. Whereas predictions 5–6 arise both from the point and compartmental model, predictions 1–4 arise specifically from the compartmental model. Testing some of these predictions no doubt would require *in vitro* techniques, which might require adapting the procedures in order to permit spontaneous activity in the perforant path axons so that heterosynaptic LTD can be elicited.

## Methods

### Compartmental model of the granule cell

We used the NEURON simulation program (version 7.3, [48, 49]). Compartmental simulations were performed using an established active model of the hippocampal dentate gyrus granule cell with detailed biophysical properties previously published by Aradi and Holmes [28] and modified by Santhakumar et al. [29]. Simulation files were downloaded from the ModelDB database at http://senselab.med.yale.edu/modeldb/, accession No. 51781. The model granule cell comprised five distinct sections [29] i.e. soma, granule cell layer dendritic section, proximal, middle, and distal dendritic sections. The total number of segments was 125, i.e. one segment for soma (length L = 16.8 um), 2x5 segments for the two granule cell layer dendritic sections (L = 50 um), 2x19 segments for the two inner molecular layer dendritic sections, the middle molecular layer dendritic sections which contain the medial path synapses, and the outer molecular layer dendritic sections which contains the lateral path synapses (each section had L = 150um) [50]. The sections contained nine voltage-activated channels: sodium, fast and slow delayed rectifier potassium, A-Type potassium, T-, N-, L-Type calcium, calcium-dependent SK- and BK-channels [29]. Parameters for passive and active properties were taken from Santhakumar et al. [29]. The channels were modelled using biologically realistic densities and kinetics. The model granule cell was able to reproduce firing behavior and basic physiological values (resting membrane potential, input resistance, membrane time constant, action potential threshold and amplitude, afterhyperpolarization, spike frequency adaptation and sag ratio) determined from electrophysiological measurements [29, 51]. Excitatory synaptic conductance changes were simulated using the sum of two exponential functions: rise time 0.2 ms; decay time 2.5 ms; reversal potential 0 mV. The peak synaptic conductance (representing synaptic weight) was modified according to the plasticity rule described in the next subsection and implemented in the custom-written NEURON.mod file.

### STDP with fast metaplasticity/homeostasis

To simulate synaptic plasticity, we employed the STDP rule modified to incorporate metaplasticity (fast homeostasis) as in [12]. For the STDP rule we used a pair-wise formula experimentally documented in hippocampal granule cells [38]. Taking into account the theoretical results with respect to the spike interaction scheme and relationship to the BCM theory [23], we simulated the so-called presynaptic centered nearest-neighbor implementation of STDP [52]. That is, for each presynaptic spike, only two postsynaptic spikes are considered; the one that occurs before and the one that occurs after the presynaptic spike, i.e.:

$$w(t+\;\delta t)\;=\;w\left(t\right)\;(1\;+\;\Delta w_{p}\;-\;\Delta w_{d})$$ (1)

We had two main reasons for choosing a nearest-neighbor STDP rule instead of an all spike interaction rule. 1. Izhikevich and Desai [11] have shown that whereas all-to-all spike pairing rule fails to make STDP compatible with classical BCM formulation of LTP/LTD, nearest-neighbor pairing rule is able to link STDP to classical LTP/LTD (see e.g. their Fig 1D and 1F). In other words, a classical BCM-like LTP/LTD curve (BCM LTP/LTD frequency threshold) emerges only from nearest-neighbor STDP rules but not from all-to-all spike interactions. Thus, since our LTP/LTD model is based on STDP and BCM, we had to use a nearest-neighbor STDP rule. 2. Our biological justification for using a nearest-neighbor STDP rule is that neurons do not seem to possess plausible biophysical mechanisms which would allow them to store all preceding spikes in their memory for tens of minutes. Izhikevich and Desai [11] mention an additional biological reason to consider only nearest-neighbor pairs: the most recent backpropagating spike overrides the effect of all the earlier spikes by resetting the membrane voltage in the dendrite.

In the pair-wise implementation of STDP, the presynaptic spikes that precede (follow) postsynaptic spikes within a certain time window produce long-term strengthening (weakening) of synapses, respectively. Thus, the positive and negative synaptic changes, Δw_p_ and Δw_d_ are calculated according to formula:

$$\Delta w_{\text{p}}(\Delta t)\;=\;A_{\text{p}}\text{ exp}(-\Delta t\;/\;\tau_{\text{p}})\text{ if }\Delta t\;>\;0$$ (2)

$$\Delta w_{\text{d}}(\Delta t)\;=\;A_{\text{d}}\text{ exp}(\Delta t\;/\;\tau_{\text{d}})\text{ if }\Delta t\;<\;0$$ (3)

where Δ*t* = *t*_post_—*t*_pre_, is due to a single pre- and postsynaptic pair [38]. Parameters A_p_ and A_d_ determine the amplitude of synaptic change, which occurs when Δt is close to zero, while τ_p_ and τ_d_ determine the time windows over which synaptic changes can occur.

In the compartmental model, however, t_post_ is the time when the postsynaptic voltage at the site of synapse crosses a dendritic voltage threshold of -37 mV, which is an important parameter of our model. Thus, in the compartmental model we should rather speak about pre-post event-pairing timing (ETDP) rule, in which the presynaptic event is a presynaptic spike, but the postsynaptic event is the local voltage crossing a given threshold. This threshold is the same for synaptic depression and potentiation, and may correspond to a membrane voltage at which the magnesium block is removed from NMDARs, as we know that both LTD and LTP in granule cells are NMDAR-dependent.

Benuskova and Abraham [12] proposed that the amplitudes of positive and negative synaptic changes, A_p_ and A_d_, are not fixed, but instead they dynamically change as a function of the average of the postsynaptic spiking activity over some recent past ⟨c⟩ such that at each time instant *t*, i.e.:

$$A_{\text{p}}\left(t\right)\;=\;A_{\text{p}}\left(0\right)\;/\;\langle \text{c}\rangle$$ (4)

$$A_{\text{d}}\left(t\right)\;=\;A_{\text{d}}\left(0\right)\;\langle \text{c}\rangle$$ (5)

Positive constants A_p_(0) and A_d_(0) are initial amplitude values for synaptic potentiation and depression, respectively. Eqs (4) and (5) simply mean the amplitude for LTP gets smaller and the LTD amplitude gets larger when the average postsynaptic activity is high. The opposite is true for a low average postsynaptic activity. Then, it is easier to potentiate the synapses than to weaken them due to an expanded amplitude for LTP and shrunken amplitude for LTD. The new values of A_p_(t) and A_d_(t) are updated at each iteration based on the current value of the average activity ⟨c⟩. Average postsynaptic activity ⟨c⟩ is calculated as an integral:

$$\langle c\rangle =\frac{\alpha }{\tau }\int_{-\infty }^{t}c(t^{\prime })\text{ exp}\left(\frac{-(t-t^{\prime })}{\tau }\right)dt^{\prime }$$ (6)

with *c*(*t*’) = 1 or 0 if the postsynaptic spike is present or absent at time t, respectively, τ is the integration period, and α is the scaling constant. We used the value of τ = 60s for all the simulations and α = 2500 when the integration step was 0.2 ms and α = 5000 when it was 0.1 ms. The averaged postsynaptic activity (Eq 6) expresses the weighted average of the postsynaptic spike count, with the most recent spikes entering the integral with bigger weight than previous ones. The integral can be replaced with a discrete sum [14], but we numerically calculated the above integral in our code. We did not employ any weights renormalization, but we did set a hard upper bound of 100% synaptic weight change. 100% change was however reached only when the lateral spontaneous spiking was turned off. The rationale for using the spike count for (Eq 6) comes from experiments of Abraham et al. [20], in which antidromic spikes (with NMDA receptors blocked) were sufficient to increase the threshold for subsequent LTP induction by HFS. The particular integral (Eq 6) was inspired by the calculation of the dynamic position of the LTD/LTP threshold in the plasticity model of the visual [5] and somatosensory cortices [6, 7].

### Simulation of spontaneous presynaptic activity in the compartmental model

Medial and lateral synaptic inputs were activated by presynaptic spikes generated by independent spike generators (NEURON’s built-in point process NetStim). In NEURON, the inter spike interval (*ISI*) of spiking activity is generated according to the following formula:

$$ISI\;=\;(1-\;noise)ISI_{0}\;+\;neg\text{exp}(-noiseISI_{0})$$ (7)

(www.neuron.yale.edu/neuron/static/docs/help/neuron/neuron/mech.html#NetStim). When the *noise* is zero, the *ISI* is equal to the initial value *ISI*_0_ and spiking activity is fully periodic with the period equal to *ISI*_0_. When *noise* is equal to one, the spiking train is random corresponding to the homogeneous Poisson distribution. When *noise* is between zero and one, the spiking activity is quasi-periodic with the mean frequency of <1 / *ISI*>. In our NEURON code, each synapse gets an independent train of spikes with *ISI*_0_ = 125ms and noise = 0.05.

### LTP induction protocols

The 400 Hz-DBS protocol consisted of five trains of 10 pulses delivered at 400 Hz at a delta (1 Hz) interburst frequency repeated 10 times at 1 min intervals [25]. The 100 Hz-TBS had 10 trains of 4 pulses delivered at 100 Hz and a theta (5 Hz) interburst frequency repeated eight times at 10 s intervals. The 400 Hz-TBS was a “hybrid” of the two of these two protocols involving 10 trains at TBS (5 Hz) but using 400 Hz intraburst frequency, repeated eight times at 10 s intervals. All HFS protocols were superimposed upon the ongoing spontaneous spiking as described above (S2 Fig).

## Supporting Information

S1 Fig Effect on the magnitude of LTP and concurrent heterosynaptic LTD when varying the percentage of tetanized medial path synapses in the compartmental granule cell model. Results for values 20%, 60% and 100%. Other values: t_p_ = 20ms, t_d_ = 70 ms, noise 0.05, A_p_(0) = 0.003, A_d_(0) = 0.001.
(PDF)

S2 Fig Effect on the magnitude of LTP and concurrent heterosynaptic LTD when varying the fractional noise of the input spontaneous spiking in the compartmental granule cell model. (A) Illustration of two different levels of noise (0.05 and 1) upon the synchrony of spikes between two randomly chosen medial and two lateral input synapses and illustration of superposition of HFS protocol (400-DBS) upon an ongoing spontaneous input activity. (B) Effect of different levels of fractional noise upon the magnitude of LTP and concurrent LTD in the compartmental model of granule cell. Other values: t_p_ = 20ms, t_d_ = 70 ms, 60% of tetanized medial synapses, A_p_(0) = 0.003, A_d_(0) = 0.001.
(PDF)

S3 Fig Effect on the magnitude of LTP and concurrent heterosynaptic LTD when varying the decay constants in the STDP rule in the compartmental granule cell model. **(A)** Results for values of t_d_ = 30 ms, 70 ms and 100 ms when t_p_ = 20ms. (B) Results for values of t_p_ = 10 ms, 20 ms and 40 ms when t_d_ = 70 ms. Other values: 60% tetanized MPP synapses, noise 0.05, A_p_(0) = 0.003, A_d_(0) = 0.001.
(PDF)

S4 Fig Effect on the magnitude of LTP and concurrent heterosynaptic LTD when changing the ratio of initial A_p_ / A_d_ in the compartmental granule cell model. Results for ratios 1: 1 and 3: 1. Other values: t_p_ = 20ms, t_d_ = 70 ms, noise 0.05, 60% of tetanized medial synapses. Ratio 3: 1 was used for the results in the main text.
(PDF)

S5 Fig Effect on the magnitude of LTP and concurrent heterosynaptic LTD when changing the dendritic threshold for detection of the postsynaptic event to be paired with presynaptic spike in the compartmental granule cell model. Results for dendritic threshold values -30 mV, -33 mV, -37 mV and -40 mV. Other values: t_p_ = 20ms, t_d_ = 70 ms, noise 0.05, 60% of tetanized medial synapses, A_p_(0) = 0.003, A_d_(0) = 0.001.
(PDF)
