# Correction: A Voltage-Based STDP Rule Combined with Fast BCM-Like Metaplasticity Accounts for LTP and Concurrent "Heterosynaptic" LTD in the Dentate Gyrus *In Vivo*

**DOI:** 10.1371/journal.pcbi.1004854

**Published:** 2016-03-22

**Authors:** The PLOS Computational Biology Staff

There is an error within the main text (Methods; 2nd paragraph) of this manuscript (p. 20 in the PDF).

The sentences read as follows:

“…tau is the integration period…” and “We used the value of tau = 60s for all the simulations…”

They should read:

“…tau is the integration time constant…” and “We used the value of tau = 12s for all the simulations…”

The following sentence should be added to the text of the Methods section (after the above cited sentences):

“In Fig 8, we used the values of tau = 1.2, 12, 120 and 240 s for the simulations, which correspond to integration time periods of 0.1, 1, 10 and 20 min, respectively.”
