# Supplementary material for: A Voltage-Based STDP Rule Combined with Fast BCM-Like Metaplasticity Accounts for LTP and Concurrent “Heterosynaptic” LTD in the Dentate Gyrus In Vivo
Source: PLoS Comput Biol. 2015 Nov 6;11(11):e1004588. doi: 10.1371/journal.pcbi.1004588 (PMC4636250; doi:10.1371/journal.pcbi.1004588)
Supplement: S1 Fig — Results for values 20%, 60% and 100%. Other values: tp = 20ms, td = 70 ms, noise 0.05, Ap(0) = 0.003, Ad(0) = 0.001. (PDF) [file pcbi.1004588.s001.pdf]

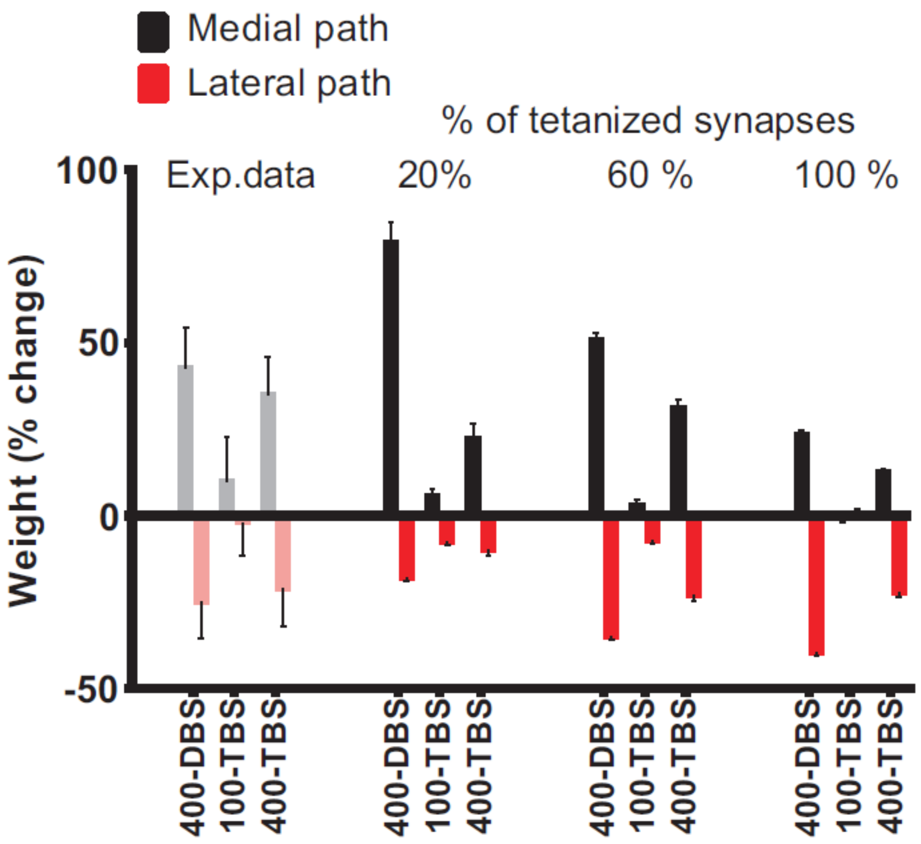

Figure S1. Effect on the magnitude of LTP and concurrent heterosynaptic LTD when varying the percentage of tetanized medial path synapses in the compartmental granule cell model. Results for values 20%, 60% and 100%. Other values: t_p = 20ms, t_d = 70 ms, noise 0.05, $A_p(0)$ = 0.003, $A_d(0)$ = 0.001.
