# Supplementary material for: A Voltage-Based STDP Rule Combined with Fast BCM-Like Metaplasticity Accounts for LTP and Concurrent “Heterosynaptic” LTD in the Dentate Gyrus In Vivo
Source: PLoS Comput Biol. 2015 Nov 6;11(11):e1004588. doi: 10.1371/journal.pcbi.1004588 (PMC4636250; doi:10.1371/journal.pcbi.1004588)
Supplement: S2 Fig — (A) Illustration of two different levels of noise (0.05 and 1) upon the synchrony of spikes between two randomly chosen medial and two lateral input synapses and illustration of superposition of HFS protocol (400-DBS) upon an ongoing spontaneous input activity. (B) Effect of different levels of fractional noise upon the magnitude of LTP and concurrent LTD in the compartmental model of granule cell. Other values: tp = 20ms, td = 70 ms, 60% of tetanized medial synapses, Ap(0) = 0.003, Ad(0) = 0.001. (PDF) [file pcbi.1004588.s002.pdf]

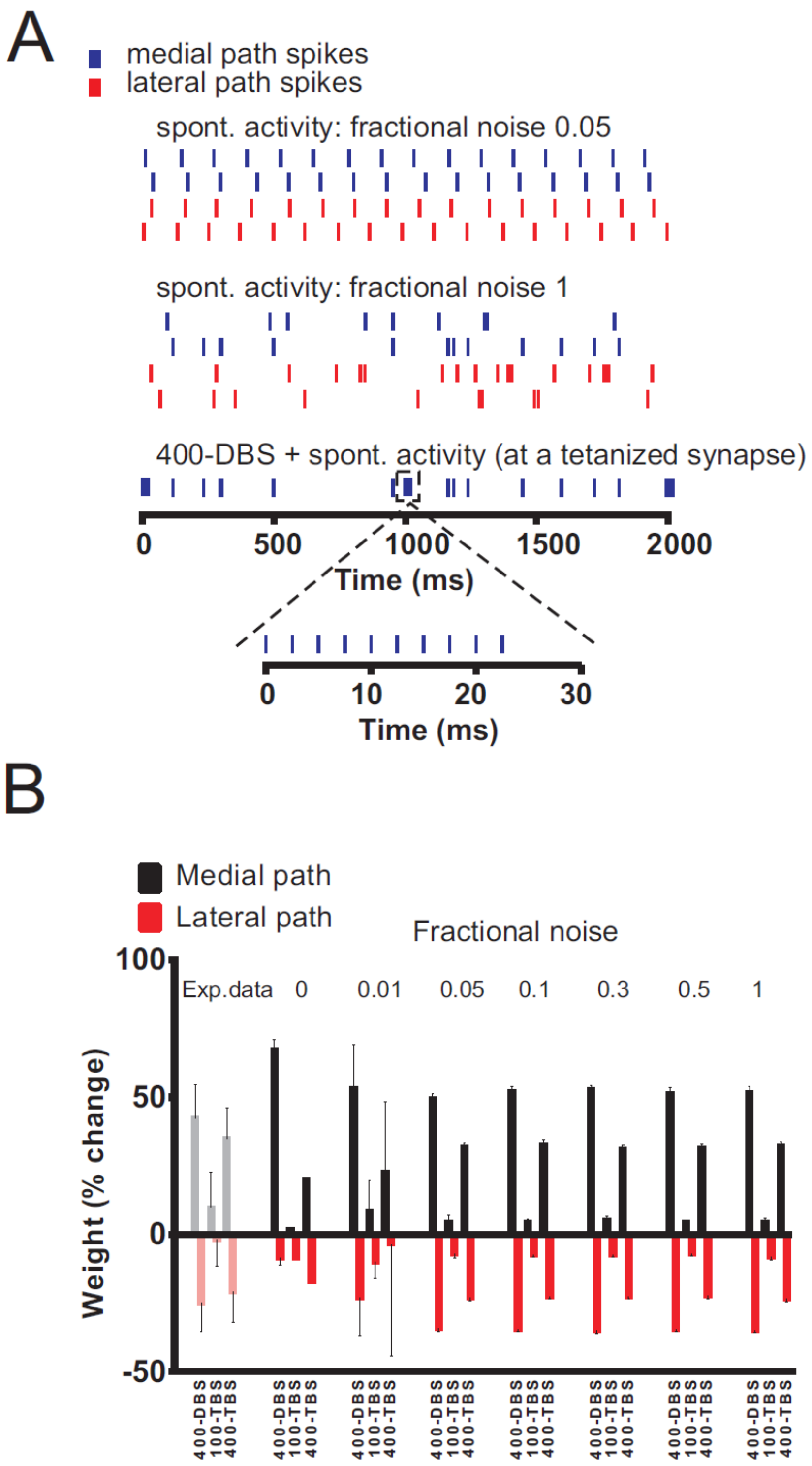

Figure S2. Effect on the magnitude of LTP and concurrent heterosynaptic LTD when varying the fractional noise of the input spontaneous spiking in the compartmental granule cell model. (A) Illustration of two different levels of noise (0.05 and 1) upon the synchrony of spikes between two randomly chosen medial and two lateral input synapses and illustration of superposition of HFS protocol (400-DBS) upon an ongoing spontaneous input activity. (B) Effect of different levels of fractional noise upon the magnitude of LTP and concurrent LTD in the compartmental model of granule cell. Other values: t_p = 20ms, t_d = 70 ms, 60% of tetanized medial synapses, $A_p(0) = 0.003$, $A_d(0) = 0.001$.
