# Supplementary material for: A Voltage-Based STDP Rule Combined with Fast BCM-Like Metaplasticity Accounts for LTP and Concurrent “Heterosynaptic” LTD in the Dentate Gyrus In Vivo
Source: PLoS Comput Biol. 2015 Nov 6;11(11):e1004588. doi: 10.1371/journal.pcbi.1004588 (PMC4636250; doi:10.1371/journal.pcbi.1004588)
Supplement: S3 Fig — (A) Results for values of td = 30 ms, 70 ms and 100 ms when tp = 20ms. (B) Results for values of tp = 10 ms, 20 ms and 40 ms when td = 70 ms. Other values: 60% tetanized MPP synapses, noise 0.05, Ap(0) = 0.003, Ad(0) = 0.001. (PDF) [file pcbi.1004588.s003.pdf]

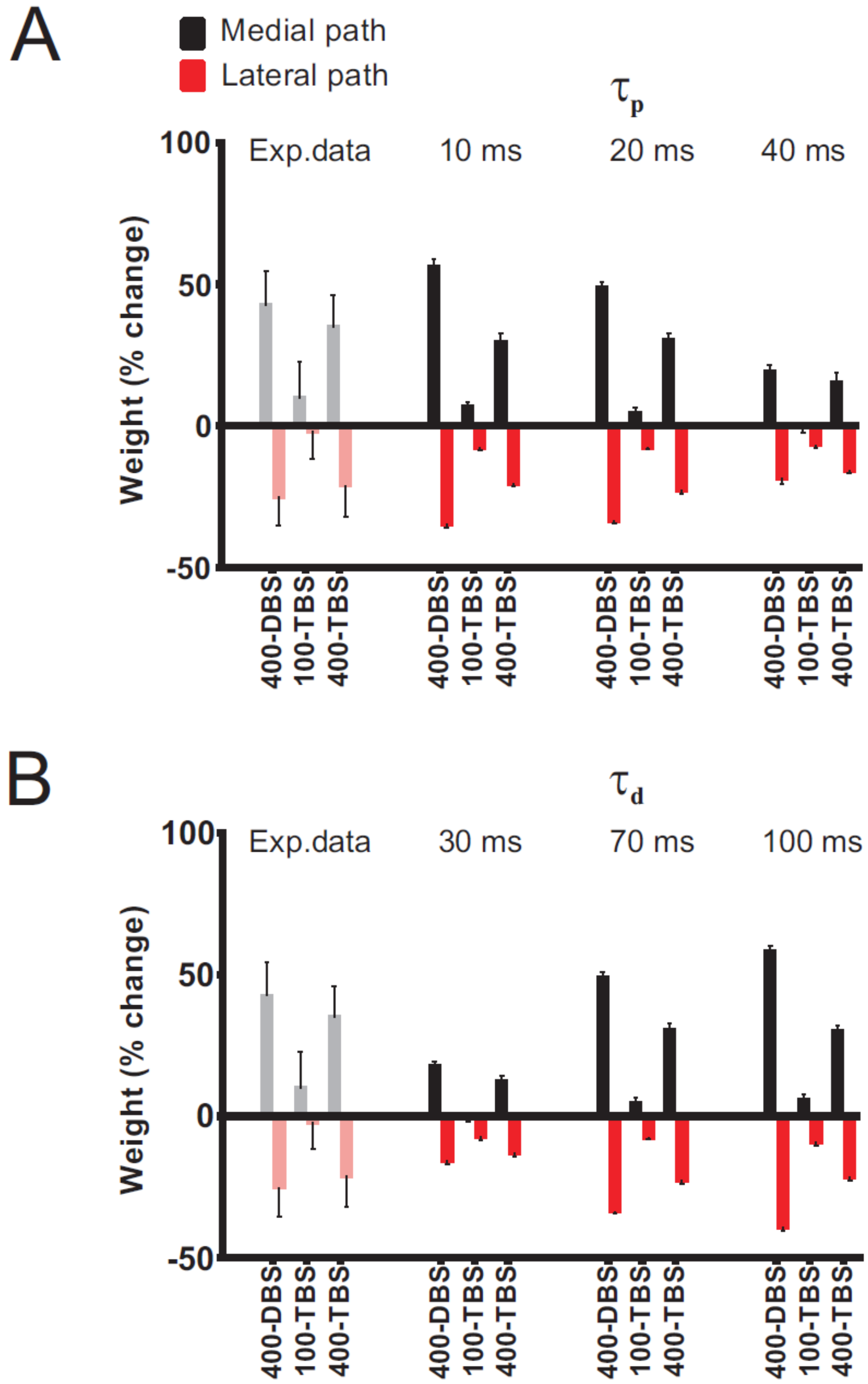

Figure S3. Effect on the magnitude of LTP and concurrent heterosynaptic LTD when varying the decay constants in the STDP rule in the compartmental granule cell model. (A) Results for values of t_d = 30 ms, 70 ms and 100 ms when t_p = 20ms. (B) Results for values of t_p = 10 ms, 20 ms and 40 ms when t_d = 70 ms. Other values: 60% tetanized MPP synapses, noise 0.05, $A_p(0) = 0.003$, $A_d(0) = 0.001$.
