# Supplementary material for: A Voltage-Based STDP Rule Combined with Fast BCM-Like Metaplasticity Accounts for LTP and Concurrent “Heterosynaptic” LTD in the Dentate Gyrus In Vivo
Source: PLoS Comput Biol. 2015 Nov 6;11(11):e1004588. doi: 10.1371/journal.pcbi.1004588 (PMC4636250; doi:10.1371/journal.pcbi.1004588)
Supplement: S4 Fig — Results for ratios 1: 1 and 3: 1. Other values: tp = 20ms, td = 70 ms, noise 0.05, 60% of tetanized medial synapses. Ratio 3: 1 was used for the results in the main text. (PDF) [file pcbi.1004588.s004.pdf]

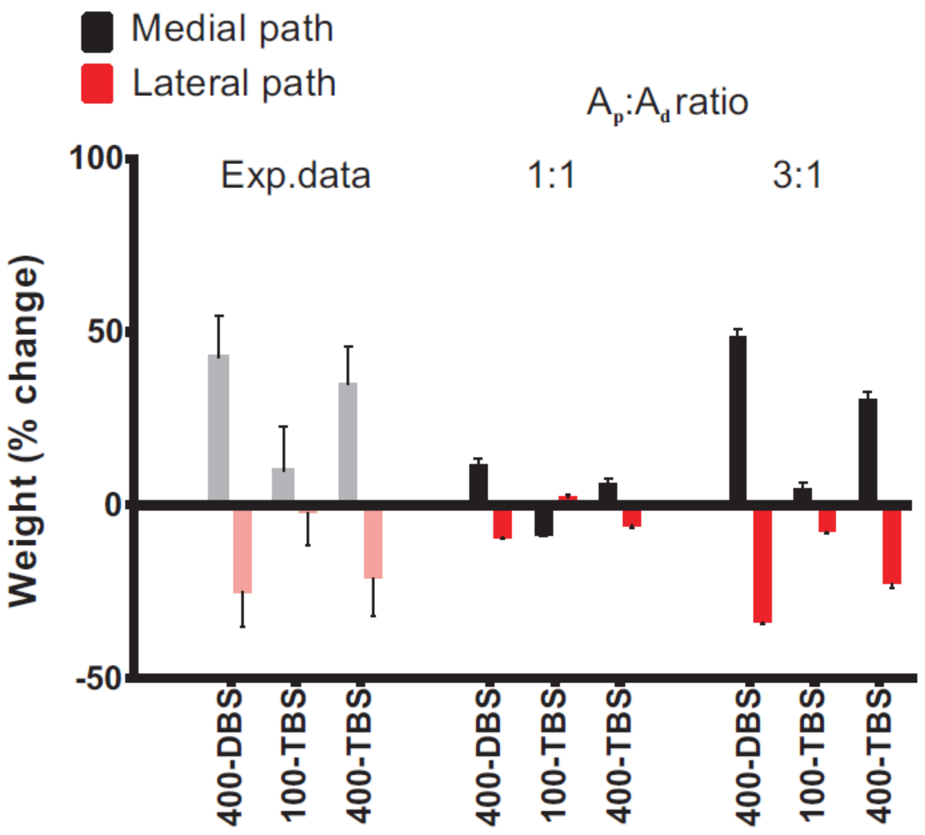

Figure S4. Effect on the magnitude of LTP and concurrent heterosynaptic LTD when changing the ratio of initial A_p / A_d in the compartmental granule cell model. Results for ratios 1 : 1 and 3 : 1. Other values: t_p = 20ms, t_d = 70 ms, noise 0.05, 60% of tetanized medial synapses. Ratio 3 : 1 was used for the results in the main text.
