# Supplementary material for: A Voltage-Based STDP Rule Combined with Fast BCM-Like Metaplasticity Accounts for LTP and Concurrent “Heterosynaptic” LTD in the Dentate Gyrus In Vivo
Source: PLoS Comput Biol. 2015 Nov 6;11(11):e1004588. doi: 10.1371/journal.pcbi.1004588 (PMC4636250; doi:10.1371/journal.pcbi.1004588)
Supplement: S5 Fig — Results for dendritic threshold values -30 mV, -33 mV, -37 mV and -40 mV. Other values: tp = 20ms, td = 70 ms, noise 0.05, 60% of tetanized medial synapses, Ap(0) = 0.003, Ad(0) = 0.001. (PDF) [file pcbi.1004588.s005.pdf]

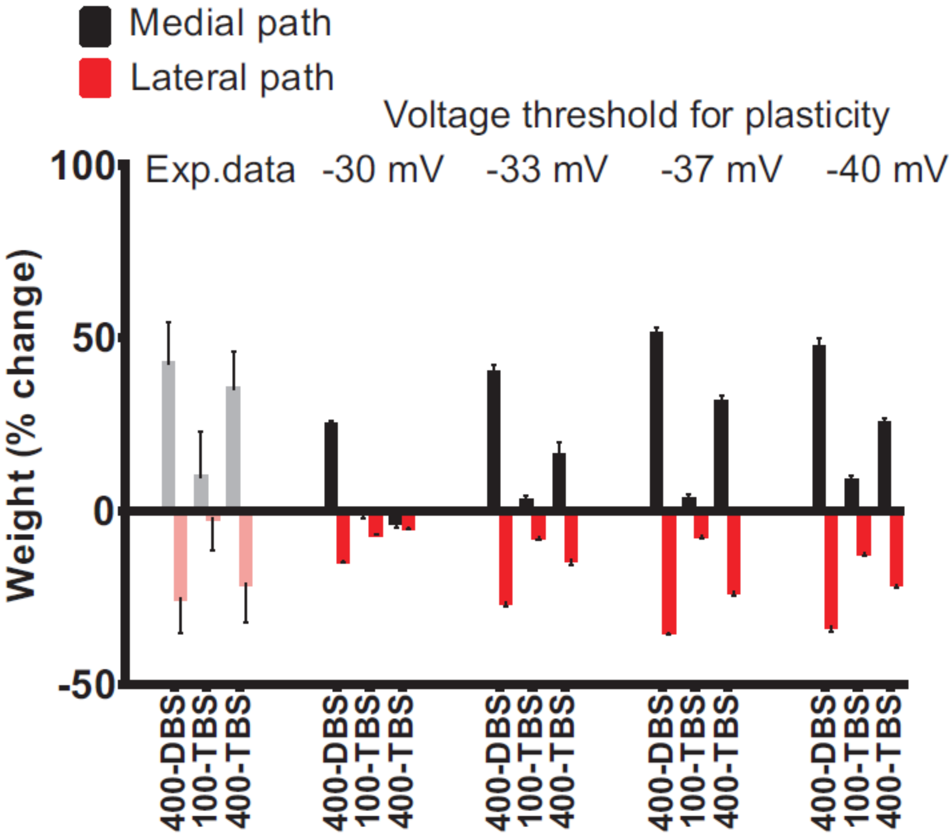

Figure S5. Effect on the magnitude of LTP and concurrent heterosynaptic LTD when changing the dendritic threshold for detection of the postsynaptic event to be paired with presynaptic spike in the compartmental granule cell model. Results for dendritic threshold values -30 mV, -33 mV, -37 mV and -40 mV. Other values: t_p = 20ms, t_d = 70 ms, noise 0.05, 60% of tetanized medial synapses, $A_p(0)$ = 0.003, $A_d(0)$ = 0.001.
